# Supplementary material for: Genome-Wide Identification and Expression Analysis of TONNEAU1 Recruited Motif (TRM) Gene Family in Tomato
Source: Int J Mol Sci. 2025 Apr 13;26(8):3676. doi: 10.3390/ijms26083676 (PMC12027651; doi:10.3390/ijms26083676)
Supplement: Supplementary file 1 [file ijms-26-03676-s001.zip › Table S1.pdf]

Supplementary Table S1: Gene descriptions and protein characteristics of 28 members of TRM gene family in tomato.

Gene Name	CDS Length (bp)	Chr	Position(5'-3')	Protein Length(aa)	Protein Charateristics					Subcellular Location
					MW (Kda)	Theoretical pI	Instability Index	Aliphatic Index	GRAVY	
SlTRM18	1971	5	63,999,696-64,003,902	656	74.318	8.49	51.2	72.38	-0.707	Nucl
SlTRM19	2973	9	520,525-526,308	990	110.921	6.12	65.84	66.57	-0.81	Nucl
SlTRM6/7/8a	2742	1	64,082,821-64,086,576	913	101.147	8.89	59.98	68.34	-0.671	Nucl
SlTRM6/7/8b	2709	1	82,628,481-82,635,145	902	100.255	6.95	51.96	66.15	-0.752	Nucl
SlTRM22	2889	2	46,910,206-46,915,171	962	106.176	8.72	55.61	73.73	-0.565	Chlo
SlTRM31	924	9	400,833-402,407	307	35.264	9.15	42.31	69.8	-0.813	Nucl
SlTRM25	2145	3	1,391,066-1,393,915	714	79.853	9.41	64.17	69.5	-0.764	Nucl
SlTRM17/20a	2880	6	46,575,297-46,580,906	959	106.938	5.78	60.2	67.97	-0.713	Pero
SlTRM17/20b	2166	9	57,148,919-57,154,322	721	80.782	5.74	60.2	71.91	-0.608	Pero
SlTRM3/4	3279	3	59,272,513-59,278,838	1092	121.657	9.26	67.04	66.08	-0.837	Nucl
SlTRM1/2/3/4/5	2196	2	48,984,063-48,988,479	732	82.563	9.68	67.19	63.55	-1.004	Chlo
SlTRM5	2403	7	3,583,904-3,588,290	800	89.805	8.94	71.9	72.96	-0.75	Nucl
SlTRM3	220	2	48,983,433-48,983,648	71	8.386	4.41	66.7	100.28	-0.72	Nucl
SlTRM30	3231	4	62,480,964-62,485,943	1076	120.795	9.07	47.76	69.87	-0.885	Nucl
SlTRM30/34b	2070	12	1,583,721-1,588,955	689	77.495	8.55	54.09	76.24	-0.701	Nucl
SlTRM30/34a	2508	7	40,860,700-40,864,471	835	94.261	9.19	45.07	76.97	-0.692	Nucl
SlTRM13/14/15/33a	2865	1	78,337,027-78,343,173	954	107.175	5.11	62.57	71.5	-0.767	Nucl
SlTRM13/14/15/33b	2445	8	62,355.476-62,359.363	814	92.691	6.51	51.49	73.66	-0.751	Nucl
SlTRM26b	2409	3	4,517,940-4,521,844	802	91.871	6.23	57.27	71.7	-0.822	Nucl
SlTRM16/32b	2199	9	4,123,568-4,126,458	732	82.58	7.31	59.46	74	-0.669	Nucl
SlTRM16/32c	2496	10	63,319,081-63,322,545	831	93.737	6.77	56.81	80.65	-0.655	Nucl
SlTRM16/32a	2145	6	44,953,004-44,956,238	714	82.002	5.31	47.81	74.1	-0.788	Nucl
SlTRM27/28a	1476	9	2,632,416-2,636,937	491	56.928	5.01	69.41	66.31	-0.861	Nucl
SlTRM27/28b	1179	10	62,482,360-62,485,633	392	44.931	4.68	72.14	77.4	-0.686	Nucl
SlTRM10/11	1413	8	61,694,228-61,698,899	470	53.037	8.26	54.88	71.32	-0.674	Nucl
SlTRM9	1224	1	77,624,586-77,626,846	407	47.24	5.08	60.28	73.51	-0.762	Nucl
SlTRM26a	2673	2	44,323,889-44,329,388	890	101.943	6.3	56.2	67.17	-0.857	Nucl
SlTRM6	345	10	5,767,027-5,767,371	114	13.294	5.36	39.86	91.4	-0.296	Nucl

Abbreviations: MW, Molecular weight; GRAVY, Grand average of hydropathicity.
